# Reflection-enhanced LIF for improved label-free classification of blood inflammation with complementary LIBS validation

**DOI:** 10.1007/s00216-026-06502-5

**Published:** 2026-04-30

**Authors:** Rania M. Abdelazeem, Z. Abdel-Salam, M. Abdel-Harith

**Affiliations:** 1 Engineering Applications of Laser Department, National Institute of Laser Enhanced Science “NILES”, Cairo University, Giza, 12613 Egypt; 2 Laser Applications in Metrology, Photochemistry, and Agriculture Department, National Institute of Laser Enhanced Science “NILES”, Cairo University, Giza, 12613 Egypt

**Keywords:** RELIF, LIBS, Inflammation classification, Blood serum, Label-free analysis, PLSR, Prozone phenomenon

## Abstract

This study introduces a reflection-enhanced laser-induced fluorescence (RELIF) technique for label-free classification of inflammation severity across three levels in blood serum. RELIF overcomes key limitations of conventional laser-induced fluorescence (LIF), particularly low fluorescence sensitivity and the prozone effect, which are frequently encountered in traditional immunoassays. In RELIF, a cuvette with a reflective surface facing the incident laser beam is used to amplify the diode-laser-pumped fluorescence signal by a factor substantially greater than in standard LIF. In parallel, complementary laser-induced breakdown spectroscopy (LIBS) measurements were performed on dried serum samples using a Q-switched Nd:YAG laser to verify elemental differences among the inflammation classes. RELIF delivered enhanced spectral resolution and sensitivity, yielding approximately 1.30× to 1.53× higher fluorescence signals than conventional LIF. While standard LIF showed reduced performance, especially for samples with severe inflammation, RELIF clearly distinguished all inflammation classes, capturing subtle spectral variations and achieving higher signal-to-noise ratios. Notably, RELIF correctly identified severe inflammation as the class with the weakest fluorescence signal, thereby avoiding the false negatives associated with the prozone phenomenon without the need for sample dilution. LIBS analysis further supported class separation through differences in Ca atomic and CN molecular emissions. Multivariate analysis using partial least squares regression (PLSR) and partial least squares discriminant analysis (PLS-DA) demonstrated high predictive accuracy and robust discrimination. Model performance was further assessed using receiver operating characteristic (ROC) curves; the RELIF-based model outperformed LIF, achieving an area under the curve (AUC) of 0.94 compared with 0.84 for LIF, indicating strong classification performance. Overall, the combined RELIF–LIBS strategy offers a rapid, cost-effective, and non-destructive tool for inflammation classification.

**Graphical abstract:**

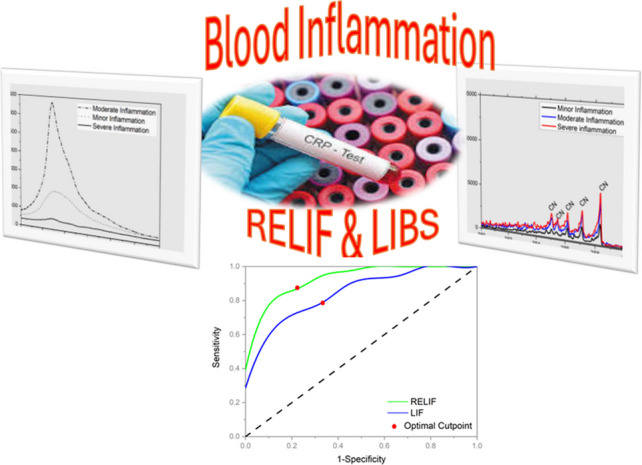

## Introduction

Inflammation is a fundamental biological response to infection, injury, or tissue damage, and it plays a central role in a wide range of diseases. These conditions cause a significant increase in morbidity and mortality, resulting in substantial socioeconomic burdens [1]. Accurately assessing the severity of inflammation is therefore essential for clinical diagnosis and for monitoring disease progression. In routine practice, however, laboratory evaluation of inflammation still relies heavily on physician expertise, which can introduce diagnostic variability and error. Conventional methods such as immunoassays and biochemical tests are widely used to measure inflammatory biomarkers, yet they often require complex sample preparation, specialized reagents, and lengthy analysis times [2].

Among blood-based biomarkers, leukocyte count, neutrophil and lymphocyte percentages, and C-reactive protein (CRP) concentration are commonly used. CRP, a liver-synthesized protein measured in serum, is particularly informative [3]. Clinically, CRP levels are often grouped into three ranges: normal/minor (0.1–<3.0 mg/L), moderate (3.0–10 mg/L), and severe inflammation (>10 mg/L) [4]. Minor elevations may be seen in conditions such as depression, obesity, diabetes, pregnancy, smoking, or genetic polymorphisms. In contrast, moderate increases are typically associated with viral or bacterial infections and major trauma. Severe elevations generally indicate acute bacterial infections. However, very high CRP concentrations (>50–100 mg/L) can lead to the prozone (hook) effect, whereby immunoassays yield falsely low or even negative results despite markedly elevated antigen levels. Performance characteristics of a point-of-care C-reactive protein assay [5]. This phenomenon has serious clinical implications, including misdiagnosis, delayed treatment, and unnecessary additional testing. Laboratories attempt to mitigate this problem through sample dilution, improved assay design [6], or kinetic measurements.

These limitations have driven interest in rapid, label-free optical diagnostic techniques that can provide biochemical information with minimal sample damage. Optical methods have already shown promise in biomedical analysis, for example, in differentiating normal and cancerous blood samples using three-dimensional reconstruction of cellular morphology from holograms [7]. Among spectroscopic techniques, laser-induced fluorescence (LIF) is particularly attractive because of its high sensitivity [8] and its ability to probe endogenous fluorophores. This technique is applied as a molecular spectrochemical analytical method in various fields, including biomedical research [9], environmental science [10], chemistry [11], biology, and medicine [12].

In a typical LIF experiment, a monochromatic laser excites the sample, and a photodetector collects the emitted fluorescence, with the liquid sample often placed in a quartz or fused-silica cuvette. However, in highly absorbing or strongly scattering biological samples, fluorescence collection efficiency can be limited, reducing diagnostic sensitivity. To overcome this, several strategies have been explored to enhance sensitivity, including the use of nanoparticles [13], quantum dots [14], and specialized optical configurations [15, 16]. In related fluorescence-based assays, nanoparticles such as gold or silver have been used to further amplify the fluorescence signal via plasmonic field enhancement [17]. However, all of these LIF enhancement approaches are complicated, costly, and, in the case of nanoparticles, invasive to the sample material.

A recent innovation by Abdel-Harith and Abdel-Salam [18] introduced reflection-enhanced laser-induced fluorescence (RELIF). This simple, label-free approach significantly advances LIF sensitivity and its potential in biomedical applications. In this method, the cuvette wall facing the excitation beam is coated with a reflective silver or aluminum layer. This mirror-like surface redirects the excitation beam back through the sample and simultaneously reflects emitted fluorescence toward the detector. As a result, both the effective excitation path length and the fluorescence collection efficiency are increased.

Blood serum contains several intrinsic fluorophores, including flavins (FAD, FMN), NADH/NADPH cofactors, aromatic amino acids, and porphyrin derivatives, which produce characteristic autofluorescence spectra [19]. Pathological changes in serum composition alter fluorophore concentrations and their microenvironments, resulting in measurable changes in fluorescence intensity and spectral features [20]. Highlighting these spectral variations enhances understanding of their diagnostic significance in biomedical analysis.

Complementary to LIF, laser-induced breakdown spectroscopy (LIBS) is a versatile optical emission technique with broad applications in metallurgy [21], pharmaceuticals [22], forensics [23], and biology [24]. LIBS enables both qualitative and quantitative elemental analysis with minimal sample preparation. A high-power Nd:YAG laser ablates the sample, creating a plasma whose atoms emit characteristic spectral lines. These emissions are collected by an echelle spectrometer coupled to an intensified charge-coupled device (ICCD) camera, facilitating elemental identification. Previous LIBS-based studies have successfully distinguished normal from inflammatory serum samples using chemometric models. Zhao et al. [2] achieved binary classification of serum samples based on LIBS features, while Abdelazeem et al. [24] combined LIF, LIBS, and chemometrics for similar classification tasks.

This study aims to enhance diagnostic capabilities in three key areas, given the recent advances. First, RELIF will be introduced to improve fluorescence sensitivity, enabling clear differentiation among three levels of inflammation: minor, moderate, and severe. This strategy also helps overcome the prozone effect that can arise in severe cases. Second, RELIF (providing molecular and fluorophore contrast) will be combined with LIBS (providing elemental and protein-related contrast) to cross-validate the resulting spectral signatures. Finally, quantitative enhancement factors and chemometric metrics will be reported to demonstrate reliable, reproducible discrimination across inflammation classes, thereby supporting more confident, consistent diagnostic decisions.

## Materials and methods

### Specimen collection and preparation

The blood serum samples used in this framework were collected from Al-Kasr Al-Aini Hospital, Cairo University. The protocol for these samples received ethical approval from the “NILES’ Ethics Committee” at Cairo University. To convert blood into serum, samples were collected in microcentrifuge tubes and incubated at room temperature for 15–30 min to clot. The clot was then separated by centrifuging the tubes for 10 min in a refrigerated unit. Immediately after centrifugation, the serum was carefully transferred to fresh microcentrifuge tubes using a pipette. For short-term use, collected serum should be kept at 2–8°C. For prolonged storage before analysis, temperatures of −  20 °C or below are recommended, as repeated freeze–thaw cycles can degrade many serum components.

In the current study, three distinct CRP ranges were selected to maximize spectroscopic discrimination: normal*/*minor inflammation (0*.*1–2.8 mg/L), moderate inflammation (5–50 mg/L), and severe inflammation (> 100 mg*/*L). Although standard clinical practice typically uses different thresholds, these specific ranges were chosen to study extreme cases with distinct biochemical profiles. It is worth noting that for each class, we tested 25 samples, each measured 10 times. The measurements for each class were then averaged.

## Experimental Setup

### Reflection-enhanced laser-induced fluorescence (RELIF) setup

**Fig. 1:**
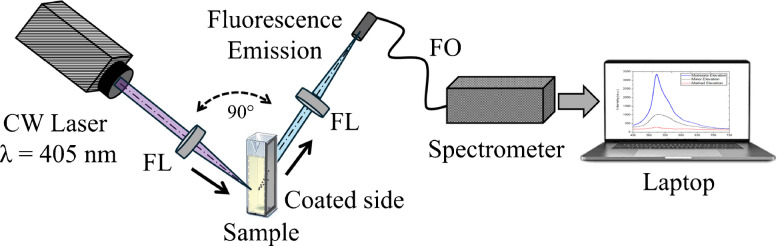
Schematic layout of the experimental setup of the reflection-enhanced laser-induced fluorescence (RELIF). CW laser, continuous wave laser; FL, focusing plano-convex lens with a focal length of 100 mm; FO, fiber optic

The experimental setup of the RELIF depicted in Fig. 1 comprises a continuous-wave (CW) laser with λ = 405 nm, a coated side cuvette, two focusing lenses (FL) with f = 100 mm, a fiber-optic (FO) cable, and a spectrometer. Changchun New Industries Optoelectronics Tech manufactures the used laser source. Co., Ltd. (CN). A 5 mW laser beam is focused on the sample in the quartz cuvette. The utilized cuvette has dimensions of 12*.*5 mm × 12*.*5 mm × 45 mm (width × depth × height). It has a special configuration in which one of its sides is coated with a thin aluminum (Al) layer deposited by a thermal coating unit (Edwards E306, UK) [18] equipped with thickness monitoring control. The coated surfaces were visually inspected and subsequently externally protected with an additional isolating layer to prevent mechanical scratches and oxidation from air exposure. The thickness of the mirror-like layer was 300 nm. The fluorescence emission was focused and collected perpendicular to the incident laser beam using an FO cable. Consequently, it was fed into a spectrometer (USB2000 FLG, Ocean Optics, USA). It was detected by a built-in CCD camera and delivered to a laptop. The commercial software SpectraSuit (OceanOptics, USA) was used to process the collected fluorescence spectra. For each diagnostic class, 25 independent samples were analyzed, with 10 technical replicates recorded per sample. These measurements were averaged to generate a representative spectral profile for each category. To ensure the reproducibility of the RELIF signal, we implemented a rigorous protocol including standardized sample cleaning and the use of a custom-engineered holder to fix the excitation and collection geometries. Furthermore, laser stability and ambient environmental conditions were monitored continuously throughout data acquisition to mitigate experimental drift.

### LIBS setup

**Fig. 2:**
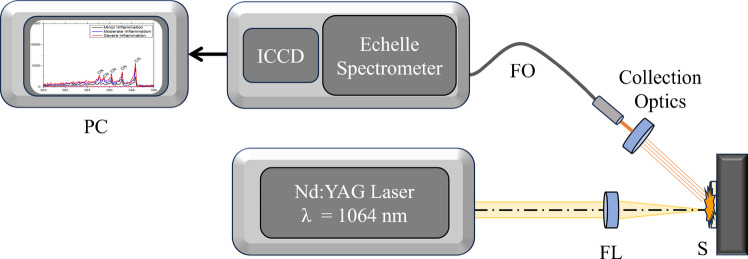
Schematic layout of the experimental setup of the laser-induced breakdown spectroscopy (LIBS). FL, focusing plano-convex lens with a focal length of 100 mm; FO, fiber optic; S, sample; ICCD, intensified charge-coupled device

Figure 2 shows the schematic layout of the LIBS experimental setup; additional technical details are provided in [25]. In brief, the system uses a Q-switched Nd:YAG laser (Brio, Quantel, France) operating at 1064 nm, delivering 50 mJ, 5-ns pulses at a repetition rate of 20 Hz. The optical path includes a focusing lens (FL), signal-collection optics, and an echelle spectrometer coupled to an intensified charge-coupled device (ICCD) camera for high-resolution spectral acquisition.

The blood serum samples were distributed onto ashless filter paper (thickness 176 µm, Whatman, Maidstone, UK) until absorbed and then left in dry air for approximately 15 min to ensure complete absorbance on the paper. The laser pulses were focused onto the blood serum sample using a plano-convex lens of a focal length of 100 mm. The sample position was controlled via an XY micrometric translation stage. The laser pulses were delivered as a single pulse ten times, with a 20 µm distance between adjacent pulses. This process was repeated in five locations on the sample surface to collect 50 spectra. The collected 50 spectra were averaged to compensate for surface inhomogeneity. A fiber optic (FO) with a length of 2 m and a core diameter of 600 µm was used to collect the emitted light from the laser-induced plasma. The FO was fed into the entrance slit of an echelle spectrometer (Mechelle 7500, Multichannel Instruments, Sweden), which was connected to an ICCD camera (DiCAM-Pro, PCO, Computer Optics, Germany) as the detector. An optimal gate width of 2500 ns and delay time of 1500 ns were used to limit continuum emission when collecting the spectra. Spectral treatment and identification of spectral lines of different elements were performed using commercial software LIBS++ [26].

## Results and discussion

### Clinical findings

In the current study, blood serum samples were measured using a fully automated clinical chemistry analyzer (Beckman AU480, USA). It is based on the turbidimetry measurement technique [27]. This is an analytical technique that estimates the turbidity, or cloudiness, of a solution by measuring the degree of light attenuation as it passes through the solution. The system measures the amount of light scattered or absorbed by the particles suspended in the solution. The results disclose three distinct classes based on estimated CRP values, each comprising 25 samples. Normal*/*minor inflammation is characterized by CRP values between 0*.*1 mg*/*L and 2*.*8 mg*/*L. The moderate inflammation class has CRP values between 5 mg*/*L and 50 mg*/*L. The severe inflammation class has CRP values > 100 mg*/*L. The collected samples were for females aged 44 to 47. Restricting the demographic range minimized biological variability related to age and gender. This enabled us to ensure that the spectroscopic differences observed were mainly related to inflammation severity.

It should be noted that the CRP ranges were intentionally selected as 0*.*1–2*.*8 mg*/*L (minor), 5–50 mg/L (moderate), and > 100 mg*/*L (severe) to maximize spectroscopic discrimination and to study extreme cases with distinct biochemical profiles. Importantly, selecting the two higher classes (moderate and severe) to test RELIF’s ability to overcome the prozone effect, which is common at concentrations above 50–100 mg*/*L.

### Spectroscopic investigations using LIF and RELIF

**Fig. 3:**
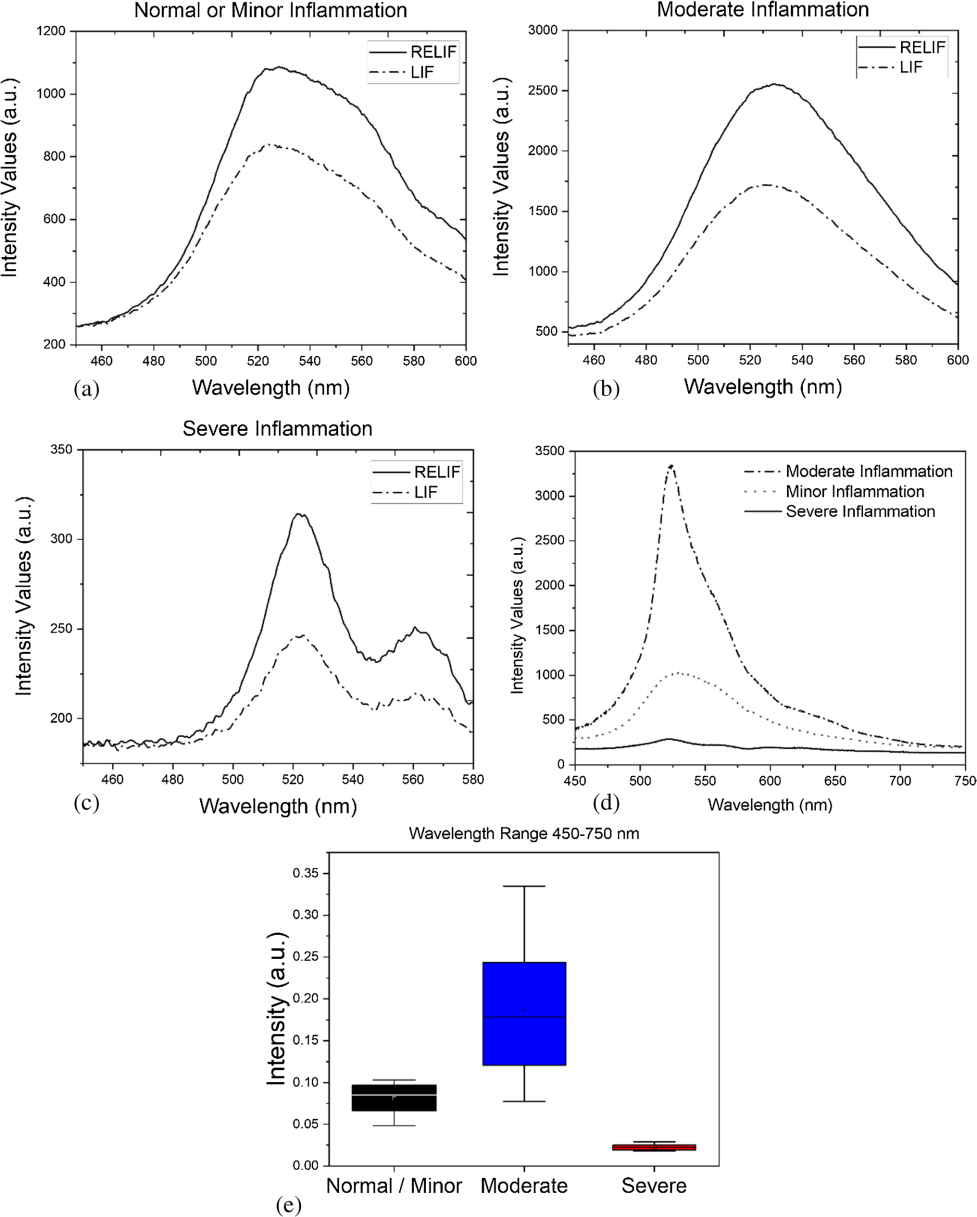
A comparison between the LIF and the RELIF spectra for (**a**) normal*/*minor inflammation, (**b**) moderate inflammation, and (**c**) severe inflammation. (**d**) The three classes, and (**e**) box chart plot of intensity for the three classes

A total of seventy-five blood serum samples were analyzed using both LIF and RELIF. Each sample was measured ten times, and the resulting spectra were averaged. All measurements were performed with excitation at 405 nm. Figure 3(a), (b), and (c) present the recorded fluorescence signals obtained by LIF and RELIF for the three sample classes. Overall, the RELIF technique increased fluorescence signal intensity by 1.30-fold in the normal/minor inflammation group, 1.53-fold in the moderate inflammation group, and 1.29-fold in the severe inflammation group.

Figure 3(d) compares the fluorescence responses of the three inflammation classes. The data indicate that fluorescence signal intensity varies markedly with the degree of inflammation. Moderate inflammation exhibits the strongest fluorescence signal, with a peak intensity at 518 nm, whereas the normal/minor inflammation class shows a weaker signal than the moderate class. This trend can be explained by changes in the concentrations of fluorescent biomolecules in blood serum as inflammation progresses. The peak near 518 nm is primarily attributed to flavins (FAD/FMN) and oxidized lipopigments. During inflammatory oxidative stress, the NADH-to-flavin (redox) ratio shifts, which accounts for the depressed fluorescence spectrum observed in the severe inflammation group [19]. In severe inflammation, a CRP concentration of more than 100 mg*/*L is characterized by a drastic reduction in the fluorescence signal. This could be attributed to multiple mechanisms: (i) Accumulation of endogenous quenching agents at higher CRP concentrations (i.e., > 100 mg/L). (ii) Protein aggregation (high-concentration CRP and acute-phase proteins) forms aggregates that reduce the concentration of fluorophores and increase light scattering. This, in turn, leads to attenuation of both excitation and emission photons. It is worth noting that the measurements were performed on undiluted serum samples to a standardized working volume in the cuvette, and raw spectra were compared across the three classes under identical conditions.

Figure 3(e) presents a box chart plot of intensity (a.u.), which helps standardize measurements and reduce variability. The box plot clearly distinguishes between the three classes based on intensity in the wavelength range 450–750 nm. The results reveal that the normal/minor inflammation class has a relatively narrow intensity range, the moderate class shows a significantly broader range, and the severe class exhibits the lowest intensity. The prozone effect can be effectively overcome by employing reflective enhanced laser-induced fluorescence (RELIF), a non-immunological, label-free technique. Unlike traditional immunological methods, RELIF mitigates this effect through its unique optical configuration, featuring a cuvette with a mirror-like reflective surface. This surface reflects the residual excitation laser beam through the sample, effectively doubling the excitation path length and increasing the probability of fluorophore excitation. This mechanism compensates for signal loss by re-exciting the sample and recapturing photons that would otherwise be lost to self-absorption or turbidity in dense serum samples. Consequently, the signal-to-noise ratio is sufficiently enhanced to detect samples with severe inflammation, which typically yield the weakest signals in standard LIF configurations. By doing so, RELIF prevents the false-negative outcomes associated with standard LIF [20]. This portable, cost-effective system, integrating a disposable reflective cuvette with a compact continuous-wave (CW) laser and fiber-optic detection, enables real-time, point-of-care classification of blood serum in clinical settings.

### Receiver operating characteristic (ROC) curves

**Fig. 4:**
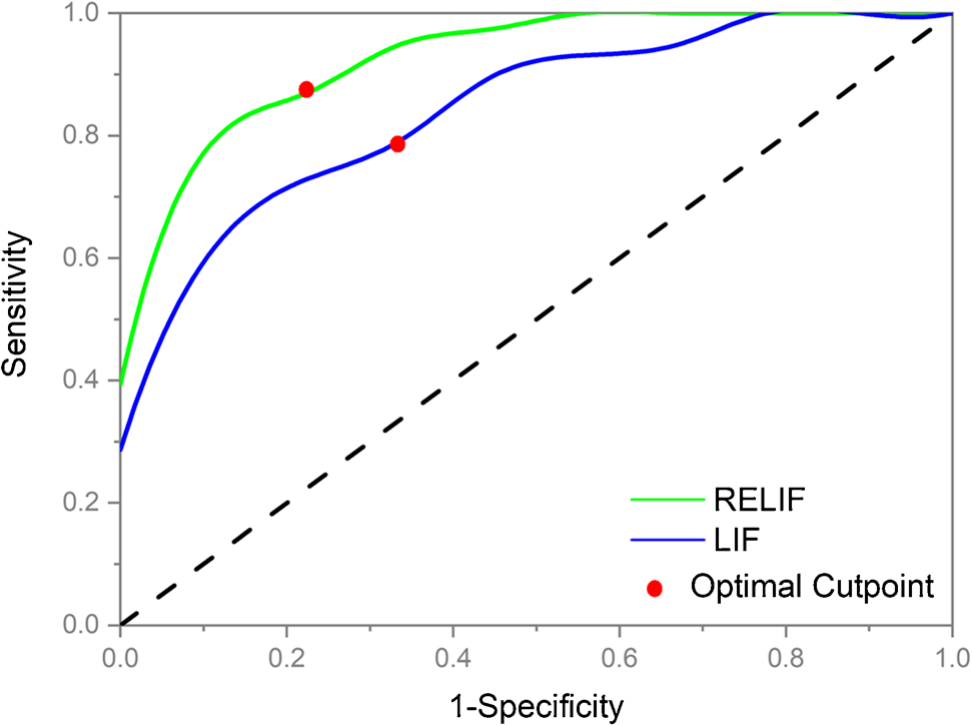
ROC analysis of RELIF (green) vs. LIF (blue) for inflammation severity classification. RELIF demonstrates superior diagnostic performance with a higher area under the curve (AUC) compared to standard LIF. Red markers indicate optimal cutpoints; the RELIF model achieves ~80% sensitivity with a significantly lower false-positive rate

To evaluate the predictive performance of the learning models, receiver operating characteristic (ROC) curves were used. These curves illustrate the trade-off between sensitivity and specificity, providing clear insight into how well each model distinguishes among healthy individuals (normal), moderate patients, and severe patients. Overall model performance was assessed by examining sensitivity, specificity, true positive rate, and true negative rate, and by considering how these metrics relate to one another. The area under the curve (AUC) was then used to summarize each model’s classification performance numerically, with values approaching 1.0 indicating excellent discrimination between positive and negative cases [28]. As shown in Fig. 4, the AUC values for each model reflect their capacity to separate normal, moderate, and severe classes. Among the evaluated models, RELIF and LIF performed best, with AUC scores of 0.94 and 0.84, respectively. These findings indicate that the RELIF model outperforms the LIF model, offering superior discriminatory power across the three clinical categories. It also validates the efficacy of the reflection-enhanced configuration in overcoming signal quenching and the prozone-like effects typical of highly inflamed serum.

### LIBS results

**Fig. 5:**
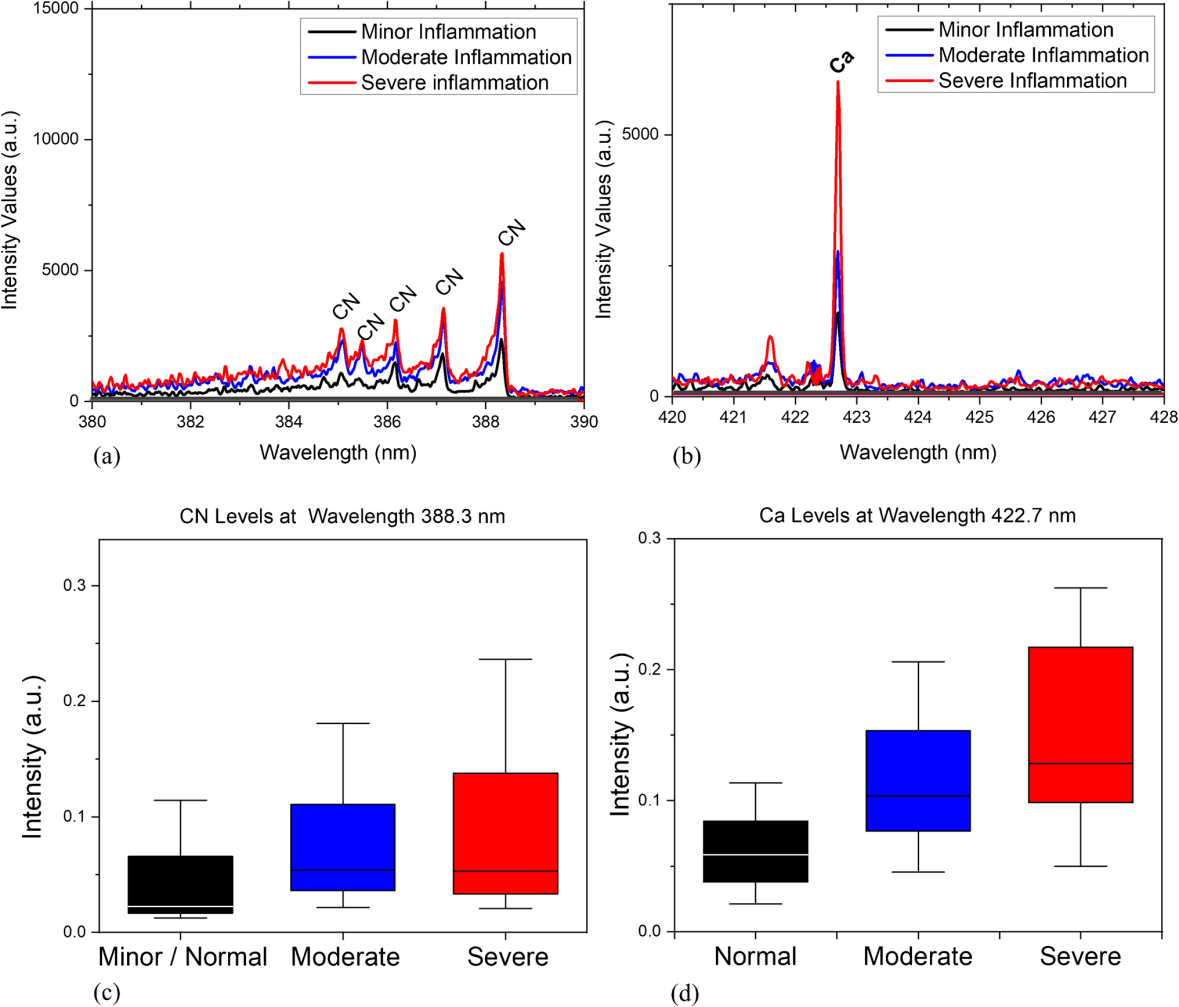
(**a**) LIBS spectra of minor, moderate, and severe inflammation classes. (**b**) Comparison between the carbon (C) 247*.*8 nm atomic line (upper), the cyanide (CN) band (middle), and the calcium (Ca) 422*.*8 nm atomic line (lower) for the three inflammation classes. (**c**) and (**d**) Box charts of the CN and the Ca elements for the three classes

Figure 5(a) shows the LIBS spectra for the minor, moderate, and severe inflammation classes, with each spectrum representing the average of 25 measurements. Figure 5(b) highlights key spectral features of carbon (C), the cyanide (CN) band, and calcium (Ca) across the three classes.

The carbon emission line at 247.8 nm (upper panel) is clearly observed in the spectra of the moderate and severe inflammation classes but is essentially absent in the minor inflammation class. Likewise, the CN molecular band around 386 nm (middle panel) exhibits marked intensity differences among the three groups. Although part of the CN emission is plasma-induced through recombination of carbon and nitrogen, its intensity also reflects the overall protein and amino acid content in the serum. The Ca line at 422.7 nm (lower panel) shows similarly distinct intensity variations, aiding discrimination between inflammation levels.

The pronounced CN band in the severe inflammation spectra indicates a higher protein content compared with the moderate and minor groups. C-reactive protein (CRP), a key biomarker of inflammation, is a major contributor to this elevated protein load. Notably, LIBS-derived protein estimates exhibit good agreement with laboratory-measured CRP concentrations. The increased intensities of the C (247.8 nm) and Ca (422.7 nm) emission lines in severe inflammation further support this association. Collectively, these correlations demonstrate that LIBS can serve as a rapid, non-destructive confirmatory tool for assessing CRP levels and, more broadly, protein content in biological samples.

The quantitative relationship between spectral intensity and inflammation severity underscores the diagnostic potential of LIBS. Compared with conventional biochemical assays, LIBS offers several practical advantages, including minimal sample preparation, near-real-time analysis, and reduced per-test cost. In addition, the strong agreement between LIBS-based metrics and reference CRP measurements confirms the analytical reliability of the technique.

Finally, the bar charts in Fig. 5(c) and (d) quantitatively demonstrate the statistical significance of the differences in CN and Ca intensities. The severe inflammation class exhibits approximately 2–3-fold higher CN intensity and about 3.5-fold higher Ca intensity than the minor inflammation class. These pronounced contrasts highlight the sensitivity and precision of LIBS for distinguishing among different degrees of inflammatory burden.

#### Partial least squares regression

Partial least squares (PLS) is a multivariate statistical technique that combines the features of principal component analysis and multiple regression. It effectively reduces dimensionality while preserving the relationship between predictors and outcomes [29]. Statistical analysis of the experimental data using partial least squares regression (PLSR) was performed with the “PLS” function in OriginPro 2021. The PLSR model was optimized with three PLS components to achieve the best balance between model complexity and predictive accuracy.

**Fig. 6:**
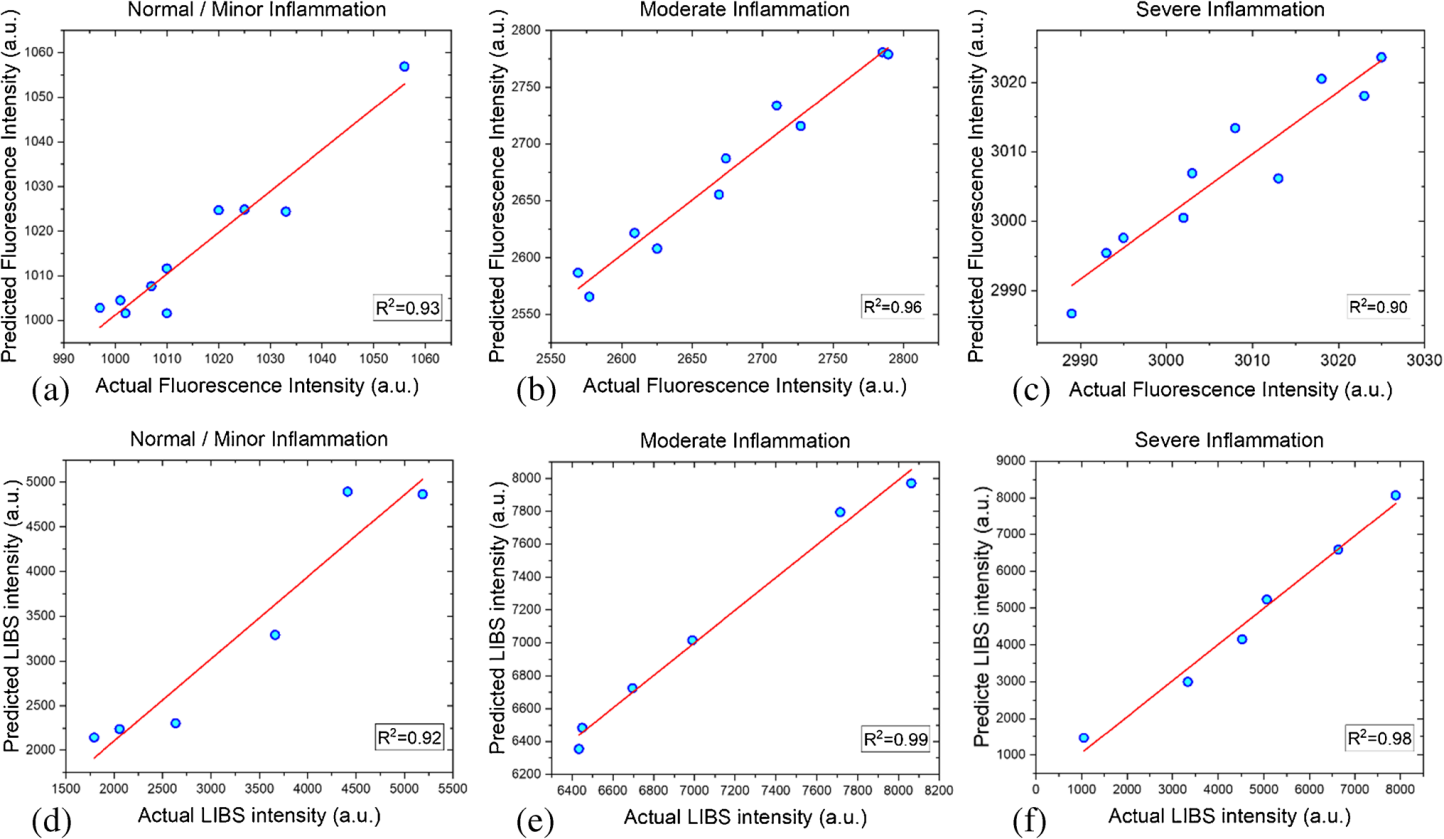
A comparison of partial least squares regression (PLSR) of LIF for (**a**) normal*/*minor inflammation, (**b**) moderate inflammation, and (**c**) severe inflammation, and of LIBS for (**d**) normal*/*minor inflammation, (**e**) moderate inflammation, and (**f**) severe inflammation. Note that each point in the graph represents the average of 10 spectra per class

The PLS model of the RELIF results shown in Fig. 6 (a), (b), and (c) demonstrates excellent performance across the three inflammation classes. The variance between the actual spectral data and the fitted predicted values yielded high coefficients of determination (R2) of 0.93, 0.96, and 0.90 for minor, moderate, and severe inflammation classes, respectively. The R^2^ value of 0*.*96 for the moderate inflammation class suggests it is the most predictable. The obtained results reveal that the RELIF spectroscopic technique can reliably predict fluorescence intensity across the three inflammation classes.

Similarly, the PLS model for LIBS results shows excellent performance across the three inflammation classes. The R^2^ values were 0*.*92, 0*.*99, and 0*.*98, respectively (see Fig. 6 (d), (e), and (f)). The exceptionally high R^2^ value of 0*.*99 for moderate inflammation suggests that this class may have the most distinct spectroscopic signature among the three classes, which is completely consistent with the PLS results of the RELIF technique. This PLS analysis demonstrates that both RELIF and LIBS techniques can reliably discriminate between different levels of inflammation severity. The high R^2^ values across the three categories suggest that the proposed techniques could be developed as quantitative diagnostic tools, potentially replacing conventional approaches for inflammation detection.

#### Partial least squares discriminant analysis (PLS-DA)

**Fig. 7:**
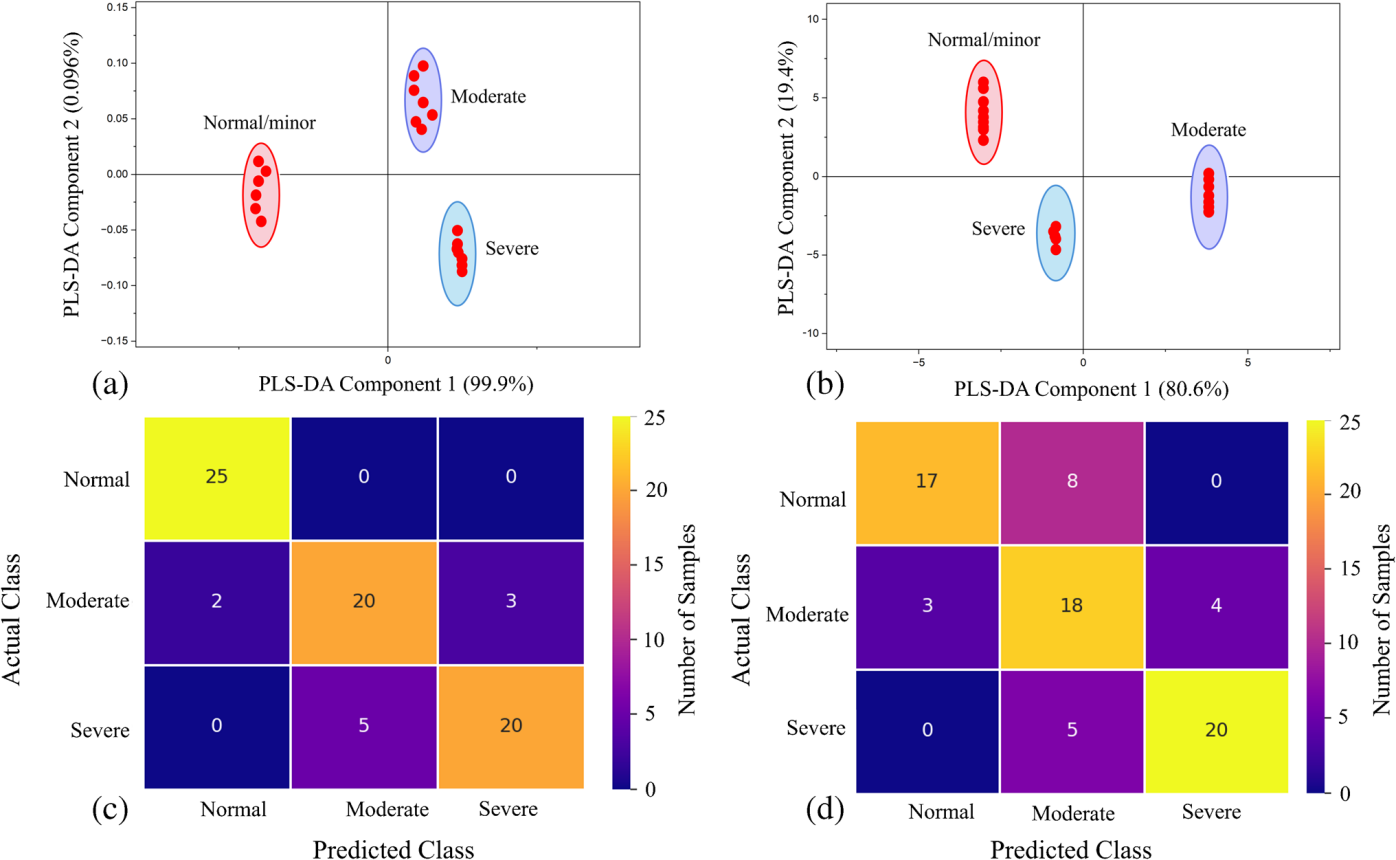
PLS-DA score plot showing clear separation of the three inflammation classes based on (**a**) RELIF spectral data with variances of component 1 and component 2 of 99*.*9% and 0*.*096%, respectively, and (**b**) LIBS spectral data with variances of component 1 and component 2 of 80*.*6% and 19*.*4%, respectively. (**c**, **d**) Confusion matrices quantifying the predictive performance of the model based on (**c**) RELIF and (**d**) LIBS, with overall accuracies of 86*.*67% and 73*.*33%, respectively

To further validate the discriminative capability of RELIF and LIBS techniques, partial least squares discriminant analysis (PLS-DA) was performed on their spectral data. PLS-DA is a supervised multivariate classification method that maximizes the separation between predefined classes by mapping the original spectral variables onto a lower-dimensional space defined by latent variables (components) [30]. Figure 7 depicts the PLS-DA score plots showing the distribution of the three inflammation classes along the first two components (component 1 and component 2). Figure 7(a) shows the PLS-DA for RELIF spectral data, with variances of 99.9% and 0.096% for components 1 and 2, respectively. Figure 7(b) depicts the PLS-DA for LIBS spectral data, with variances of 80.6% and 19.4% for components 1 and 2, respectively. The results obtained reveal superior class separation with no overlap. Each class exhibits tight grouping, indicating high similarity and strong discrimination among the other classes. The clear separation achieved by PLS-DA for RELIF spectral data confirms that its spectral features contain sufficient discriminatory information to classify the three classes reliably. This classification performance supports the clinical applicability of RELIF as a rapid, non-invasive diagnostic tool. The variance achieved by only two components indicates that the spectral differences among the inflammation classes are robust and noise-free, further validating the effectiveness of the RELIF technique. The model was validated using a cross-validation test [31], in which the dataset for each inflammation class (25 samples per class) was iteratively split into training and test subsets. In the current study, we employed leave-one-out cross-validation (LOOCV) within each class. In each iteration, one sample was withheld as the test observation, and the model was trained on the remaining samples. The prediction was made on the withheld sample. This process was repeated for all 25 samples per class.

The resulting confusion matrices (Fig. 7(c) and (d)) summarize the model’s predictive performance. For the RELIF data, the model achieved 100% accuracy for the normal class and 80% for both the moderate and severe classes, resulting in an overall accuracy of 86*.*67%. On the other hand, based on the LIBS dataset, the model yielded accuracies of 68% (normal), 72% (moderate), and 80% (severe), with an overall accuracy of 73*.*33%. These results demonstrate that while both techniques are effective, the RELIF-based model offers superior predictive performance for this specific classification task.

## Conclusions

This study demonstrates the successful application of two spectrochemical techniques, reflection-enhanced laser-induced fluorescence (RELIF) and laser-induced breakdown spectroscopy (LIBS), for classifying inflammation severity in human blood serum. RELIF, employing 405 nm excitation, produced a characteristic emission peak at 518 nm whose intensity varied systematically with inflammation stage: moderate inflammation generated the strongest signal, minor inflammation an intermediate response, and severe inflammation the weakest. These spectral trends are consistent with alterations in fluorescent biomolecule concentrations, the accumulation of quenching agents, and structural modifications of fluorophores during disease progression. The reflection-enhanced cuvette design amplified fluorescence intensities by approximately 1.30×–1.53× compared to conventional LIF, thereby improving sensitivity, enhancing the signal-to-noise ratio, and mitigating the prozone effect. Box plot analyses further confirmed distinct spectral distributions across inflammation classes, underscoring RELIF’s diagnostic robustness.

LIBS provided complementary elemental and molecular information, revealing significant differences in calcium (Ca) atomic-line and cyanide (CN) molecular-band intensities. Severe inflammation was associated with the highest Ca and CN levels, with quantitative analyses showing 2–3-fold higher CN intensity and 3.5-fold higher Ca intensity relative to minor inflammation. These elemental markers reinforced the reliability of the classification framework. Statistical validation using partial least squares regression (PLSR) demonstrated excellent predictive accuracy (R^2^ = 0.90–0.96 for LIF; 0.92–0.99 for LIBS), while partial least squares discriminant analysis (PLS-DA) confirmed clear separation among classes. receiver operating characteristic (ROC) curves further validated RELIF’s superior discriminatory performance compared to conventional LIF.

Taken together, RELIF and LIBS establish a complementary diagnostic strategy. RELIF offers rapid, label-free, and non-destructive screening with enhanced fluorescence sensitivity, making it particularly suitable for routine monitoring and preliminary assessment. Its straightforward, low-cost configuration can be miniaturized with compact lasers and optical components, enabling portable, real-time classification in clinical or primary care settings. LIBS, while more complex and resource-intensive, provides confirmatory elemental and molecular specificity, particularly for markers such as Ca and CN that correlate strongly with inflammation severity. Thus, RELIF serves as an efficient first-line screening tool, while LIBS strengthens diagnostic confidence through biochemical validation.

By integrating molecular sensitivity with elemental specificity, this dual-spectrochemical approach enables rapid classification and quantitative validation of inflammatory status. The combined framework holds considerable promise for advancing non-invasive, cost-effective, and reliable tools for diagnosing and monitoring inflammation in clinical practice.

## Data Availability

The data generated and analyzed during the current study are available from the corresponding author upon request.
